# The concept of mechanism from a realist approach: a scoping review to facilitate its operationalization in public health program evaluation

**DOI:** 10.1186/s13012-015-0345-7

**Published:** 2015-10-30

**Authors:** Anthony Lacouture, Eric Breton, Anne Guichard, Valéry Ridde

**Affiliations:** 1 EHESP French School of Public Health, Sorbonne Paris Cité, Rennes, France; 2 CNRS, UMR CRAPE Centre for Research on Political Action in Europe—6051, Rennes, France; 3 ESPUM School of Public Health University of Montreal, Montreal, Quebec Canada; 4 Faculty of Nursing, Laval University, Quebec, Quebec Canada; 5 IRSPUM University of Montreal Public Health Research Institute, Montreal, Quebec Canada

**Keywords:** Mechanism, Realist approach, Evaluation, Realism, Conceptual analysis, Public health intervention, Complexity, Context

## Abstract

**Background:**

Public health interventions are complex by nature, and their evaluation requires unpacking their intervention logic and their interactions with open social systems. By focusing on the interrelationships between context, mechanism, and outcome, Pawson and Tilley’s realist approach appears a promising innovation for public health-related evaluation works. However, and as expected of any methodological innovation, this approach is being constructed gradually by answering the multiple challenges to its operationalization that fall in its path. One of these challenges, users of this approach agree on, is the necessity of clarifying its key concept of mechanism.

**Method:**

We first collected the definitions of mechanism from published works of Pawson and colleagues. Secondly, a scoping review was conducted to identify the ones quoted by users of the realist approach for evaluating public health interventions (1997–2012). We then appraised the clarity and precision of this concept against the three dimensions defined by Daigneault and Jacobs “term, sense and referent.”

**Results:**

Of the 2344 documents identified in the scoping review, 49 documents were included. Term: Users of the realist approach use adjectives qualifying the term mechanism that were not specifically endorsed by Pawson and colleagues. Sense: None of the attributes stated by Pawson and colleagues has been listed in all of the documents analyzed, and some contributions clarified its attributes. Referent: The concept of mechanism within a realist approach can be ascribed to theory-based evaluation, complex social interventions, and critical realism.

**Conclusion:**

This review led us to reconsider the concept of mechanism within the realist approach by confronting the theoretical stance of its proponents to the practical one of its users. This resulted in a clearer, more precise definition of the concept of mechanism which may in turn trigger further improvements in the way the realist approach is applied in evaluative practice in public health and potentially beyond. A mechanism is hidden but real, is an element of reasoning and reactions of agents in regard to the resources available in a given context to bring about changes through the implementation of an intervention, and evolves within an open space-time and social system of relationships.

**Electronic supplementary material:**

The online version of this article (doi:10.1186/s13012-015-0345-7) contains supplementary material, which is available to authorized users.

## Background

Researchers working in the field of public health interventions evaluation suggest embracing their complex nature [1, 2] and their dynamic within an open system [3]. It is particularly important to understand how the intervention relates to both the individuals involved and the context in which it is implemented with the overarching aim of improving population health and reducing social inequalities in health [4]. Since the 1980s, different approaches arising out of the theory-based evaluation perspective have suggested theorizing the logic of intervention and taking into account the mechanisms through which the intervention produces its outcomes [5–8]. The realist approach, proposed in 1997 by the sociologists Pawson and Tilley [9], is increasingly attracting attention from researchers in evaluation [10, 11]. Evaluators are called to do more than just demonstrate whether the intervention works, to produce evidence on the way it works (or fails to work) in its reality, on how it produces outcomes, among whom, in what circumstances [12]. Acknowledging the richness of the context, the center of gravity for evaluation research is thus shifted away from causal intervention-outcome interplays to Context-Mechanism-Outcome configuration (CMOc) [13].

If this new approach to evaluation may at first seem innovative in public health, no consensus has emerged yet among its users with regard to the concept of mechanism, even though this is at the heart of its use [14]. Astbury and Leeuw confer three fundamental aspects upon the concept of mechanism “in line with the ‘realist’ principles: Mechanisms (1) are usually hidden, (2) are sensitive to variations in context, and (3) generate outcomes” [15] (p. 368). However, these attributes do not seem sufficiently precise for a unanimous definition of the concept within the Pawson and Tilley’s realist approach. Moreover, two systematic reviews have highlighted all the ambiguity of the concept of mechanism in its comprehension and in its use for evaluating public health interventions [16, 17]. According to these reviews, some researchers encountered difficulties in differentiating mechanisms from contextual factors, activities, or specific resources to the intervention, thus raising the issue of structuring the CMO configurations. Through what Marchal and colleagues call the CMO dilemma [18], the question of clarity of the concept of mechanism also arises. A recent article outlines the delicate exercise of the clarification of this concept in realistic evaluation using a social science illustration [19]. Through our scoping review, we have tried to do the same exercise from the practices of the realist evaluation in the field of public health. Our work has three main objectives to propose a clearer and more precise definition of the concept of mechanism: (1) trace how the concept of mechanism is defined in the writings of Pawson and his colleagues, (2) describe how this concept is defined and operationalized in the contribution of the users of the realist approach in the public health field, and (3) explain what are the differences between mechanism, intervention, and context.

## Methods

**Fig. 1:**
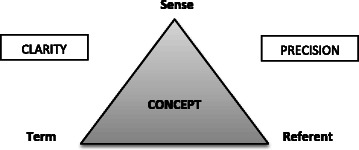
The three dimensions of a concept (adapted from Daigneault and Jacobs, 2012 [20])

To define a concept, Daigneault and Jacobs’ conceptual framework confers upon it three dimensions [20] (i.e., Fig. 1): (1) the concept is designated by a *term* (e.g., the concept of capital). A term may have several meanings (homonymy, e.g., capital as a city or as wealth) and several terms may have the same meaning (synonymy, e.g., capital and asset as resources); (2) the concept carries *sense*, which is to say that it expresses an idea (e.g., capital refers to financial resources available for use). The sense of the concept relates to the set of necessary and sufficient attributes included in this concept, an attribute being one characteristic of the concept; (3) the concept refers to a class of objects in the real world, which is designated by the *referent.* Therefore, to ensure the clarity of a concept, a term must only have a single sense within a given referent. Moreover, a concept is that much more precise when the empirical phenomena to which it applies are clearly distinct from those to which it does not apply (e.g., the concept of capital from the Marx’s economic theory or from the theory of human capital).

### Research strategies

We first collected the definitions of mechanism from the published works of Pawson and colleagues, from 1997 the year in which Pawson and Tilley’s *Realistic Evaluation* was published to 2012. This allowed us to unearth the terms and fundamental attributes characterizing this concept as well as the referents used.

Secondly, the definitions of mechanism by users of the realist approach were collected using the scoping review method. This exploratory strategy was preferred as it allows for a systematic examination of all documentation available on the concept of mechanism (i.e., scientific and gray literature) and the identification of the main gaps in the existing documentation [21, 22]. The PRISMA diagram was used to guide the scoping review process [23].

To be selected, documents had to (1) address the concept of mechanism by referring to the realist approach in evaluation, (2) deal with one (or more) public health intervention(s), (3) be written in English or French, and (4) be published between January 1997 and June 2012.

Bibliographical databases were also searched (i.e., Medline, Academic Search Complete, Eric, SAGE Journals Online, BDSP, Cairn info, and ScienceDirect). The choice of key words was adjusted according to the different thesaurus (“realist* review” OR “realist* synthes*” OR “realist* approach” OR “realist* evaluation” OR “realist* case stud*” AND “mechanism*”). We also searched the reference lists of all the articles meeting our inclusion criteria looking for other key documents (especially in the gray literature) that may have eluded our general search strategy.

The data analysis entailed (1) demonstrating how the writings collected contribute to the three dimensions of the concept of mechanism (“term, sense and referent”) as defined by Pawson and his colleagues and (2) identifying the differences in understanding and use of this concept among users of the realist approach to shed light on the conceptual gaps in the definition.

## Results

**Fig. 2:**
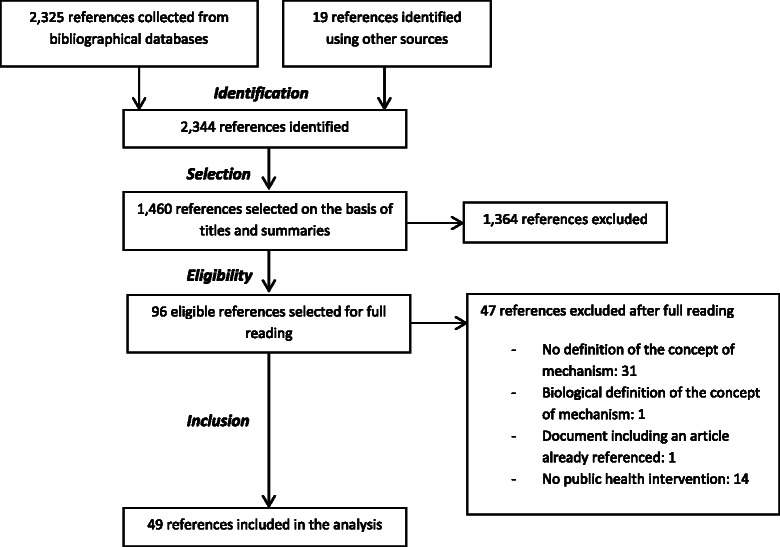
Process of the scoping review (March–June 2012) according to the PRISMA diagram

For the first step of our work, 7 references of Pawson and colleagues have been included [9, 12, 24–28] (i.e., Additional file 1). Secondly, the search for papers within the scoping review yielded 2344 references; 1460 of these were selected on the basis of their titles and abstracts. 96 documents were fully searched. 49 met the criteria for inclusion and were selected for analysis (i.e., Fig. 2).

In light of the papers collected [18, 29-76], Pawson and Tilley’s realist approach appears to be thriving. This is regardless of the level of intervention addressed (mainly that of program but also practice or policy). The approach was also applied to most areas in public health, from health system management [29, 30] to prevention and health promotion [31, 32], healthcare services [33, 34], and participative research [35, 36]. In what follows, we look at the three dimensions of the concept of mechanism (i.e., Additional file 2).

### The term of the concept of mechanism

Pawson and colleagues use the term mechanism attached to its object, “programme” or accompanied by qualifying adjectives such as “underlying,” “explanatory,” “causal,” “change,” “generative,” “intended,” “dominant,” “particular,” and “social” [9, 12, 24, 27]. Synonyms of the term mechanism are also used by the authors such as “power,” “theory,” and “force” and “trigger”.

Contrary to the synonyms of the term mechanism (e.g., “theory” [37], “force” [38], “triggers” [39]), users of the realist approach resort to adjectives not specifically endorsed by Pawson and colleagues. It should be noted that in addition to the adjectives used by Pawson and his colleagues, those chosen by users bring us back to the position of a mechanism through the different levels of the intervention contexts (e.g., “individual,” “collective,” “organizational,” “external” [40]), the nature of a mechanism (e.g., “behavioral” [40], “cognitive” [40], “emotional,” “structural” [41], “social” [42]), varying among the subjects (e.g., “participants,” “staff,” or “policymakers” mechanisms) and the relationship between them (e.g., “mechanism of,” “mechanism between”), the abstraction level of a mechanism (e.g., “causal,” “generative,” or “observed” mechanism), its emergence (e.g., “emerging” [43]), its behavior for change (e.g., “enabling,” “disabling” [44], “positive” [45], “negative” [46]), its interactivity (e.g., “interactive,” “interacting”[43]), its categorization (e.g., “primary,” “secondary,” “submechanism”), its purpose (e.g., “for change” [47] or “of change” [48]) as well as to the different components (e.g., “action,” “project,” or “program” mechanisms) and steps of the intervention to which it is attached (e.g., “implementation mechanism” [35] or “mechanism of implementability”).

### The sense of the concept of mechanism

In 1997, Pawson and Tilley conferred three fundamental attributes to the mechanism of a program [9]. It (1) reflects the embeddedness of the program within the stratified nature of social reality, (2) takes the form of propositions which will provide an account of how both macro and micro processes constitute the program, and (3) demonstrates how program outputs follow from the stakeholders’ choices (reasoning) and their capacity (resources) to put these into practice (p. 66). According to them, a mechanism is not a variable of causality *stricto sensu* but rather a theory in the sense of a logic “which spells out the potential of human resources and reasoning” (p. 68). In 2002, Pawson refined a mechanism as the subjects’ interpretation of the intervention stratagem [24]. It is not the intervention that works but the resources they offer to enable their subjects to make them work (p. 342). In this way, the intervention will work “if those subjects are persuaded to accept, install, maintain and act upon it” (p. 344). In 2004, Pawson and Tilley no longer spoke of intervention stratagem but rather of the logic of an intervention, declaring that these are the mechanisms which explain by retracing the destiny of a program theory. They also complete their definition by specifying that a mechanism is usually hidden, sensitive to variations in context, and produces effects [12]. In similar or distinct contexts, a mechanism can produce outcomes that are identical or different. In 2006, Pawson [25] added that mechanisms describe “the powers inherent in a system, be those (…) agents or structures” (p. 23), namely the “choices under the inducement of programme resources” (p. 24) to make change happen. Pawson and colleagues (2011) specified that interventions work through the reasoning and reactions (i.e., mechanisms) of its subjects [27]. In other words, mechanisms “capture the many different ways in which the resources on offer may impinge on the stakeholders’ reasoning” [28] (p. 187).

Each of these attributes has been picked up by one (or more) user(s) of the realist approach, but none of these has been listed in all of the documents analyzed. Moreover, some attributes have been deepened in the writings of users of the realist approach.

Firstly, as Pawson and colleagues, they define mechanisms (1) as reasoning (e.g., ideas) of (a) human agent(s) and their choices about how the change will be achieved through an intervention [48] and (2) as individual or collective reactions of agent(s) to the resources provided by the intervention that trigger change [49]. For instance, Robert focuses on the conviction or reticence of the staff and the satisfaction of users (i.e., mechanisms) when the exemption programs of direct care payments have been implemented in different African countries [40].

Secondly, mechanisms (i.e., reasoning and reactions of human agents) can evolve in a range of circumstances at different times [44] and according to problematic situations on which to intervene [41]. During the intervention process, including implementation, some existing strategies can die or change direction and new ones can emerge [50]. Furthermore, some users specify also that a mechanism is latent and reveals itself in the implementation of the intervention [51]. It illustrates thus the history of an intervention before its implementation. So, when an intervention is designed and implemented in a given context, mechanisms may not be triggered intentionally by the intervention designers [47].

Thirdly, even though according to Pawson and Tilley a mechanism is generally hidden, users insist on specifying that, though not directly observable, the mechanism is real [52] and exists activated or not. Although they cannot be measured directly “because they happen in people’s heads” [53] (p. 92), mechanisms, once activated in a specific context, can be identified and measured through their undesired or desired outcomes [34], making explicit one (or more) CMO configuration(s).

Finally, some users have emphasized that mechanisms are interactive with one another, with the context [54], or with the outcomes they produce [45]. These interactions can lead to positive or negative feedback loops (e.g., after negotiation between stakeholders, interference with other interventions), which may or may not lead to the success of the intervention resulting in a change or not [45, 54].

Mechanisms described by users of the realist approach for evaluating interventions in public health are mainly linked to the participation, collaboration, partnership, or management processes of/between subjects involved in the implemented intervention (e.g., stakeholders, policymakers, health workers, patients) in order to improve (or change) behaviors, practices, programs, policies, or performance of an organization (e.g., the access to quality care) in a given context. The mechanisms (i.e., reasoning and reactions of human agents) are diverse but recurring, such as “promoting reflection” [53], “taking control” [43], “increasing motivation, interest and satisfaction” [32, 53, 55], “building and increasing confidence” [43, 53, 55], “promoting mutual support” [53], “gaining acceptance of new information” [47], “creating a sense of belonging and respect” [47], or “subjects empowerment” [32].

### The referent of the concept of mechanism

The realist approach can be ascribed to the theory-based evaluation, complex social interventions, and critical realism.

### Theory-based evaluation

Firstly, the works of Pawson and colleagues arise from the proponents of theory-based evaluation approach such as Chen and Rossi [6, 7] and Weiss [5] in particular. This approach stresses the importance of making explicit the logic of the intervention (or program theory) and to clearly distinguish it from the logic of the intervention implementation. The program theory (or logic of intervention) is defined as the set of hypotheses that explains how and why the intervention is expected to produce its effects [17]. With the realist approach, a special attention is paid to the collective or individual reasoning and reactions of human agents (i.e., mechanisms) depending on the resources available which allow or hamper the change in a specific context [41]. Declined as CMO configurations constructed as part of a realist synthesis or realist evaluation, the program theory identifies and describes how mechanisms (e.g., actors’ ideas or choices) led to the outcomes for change in a given context [30, 48]. For evaluators, understanding of how the actors generate the outcomes for change in regard to the available resources is essential for a proper understanding of mechanisms searched for by realist evaluation or realist synthesis [41].

### Complex social interventions

Some users of the realist approach in evaluation [17, 33, 40] have described the several characteristics of a complex social intervention stated by Pawson [26, 27]. These interventions are long sequences of theories (i.e., a long chain of decision-making processes involving sequences of mechanisms as reasoning and reactions of human agents) in the sense of logics of intervention. As illustrated by Pawson [25], intervention theories “begin in the heads of policy architects, pass into the hands of practitioners and, sometimes, into the hearts and minds of subjects” (p. 28). As the product of the several layers of its context and involving the participation of numerous stakeholders (that are rooted in different localities, institutions, cultures, and histories [27]), these interventions are embedded in multiple social systems (i.e., systems of social relationships). They are also learning systems and prone to be borrowed, for instance to improve the delivery of interventions. According to Pawson, social complex interventions are nonlinear and sometimes go into reverse because of feedback negative or positive loops in interventions implementation. Finally, these interventions are open and dynamic systems in space and time and depend on its history and its past, being the offspring of previous interventions.

### Critical realism

The critical realism is one of the important pillars on which rests the realist approach of Pawson and Tilley [9]. The latter recognize the stratified nature of the social world and identify the generative mechanisms underlying a stratified social reality. Wilson and McCormack refer to three specific domains presented by the critical realist Bhaskar: the domain of the real (the causal mechanisms), the domain of the actual (the intervention itself), and the empirical domain (the change that is observable in reality) [56]. The evaluation of an intervention allows for the uncovering and analysis of causal mechanisms operating at the level of the real. “The prerequisite is to look beneath the surface in order to inspect how they work” ([25], p. 24). Another principle to which certain users refer like Pawson and colleagues is the generative causality. In similar or different contexts, individuals can make similar choices in such a way that reoccurring models can emerge, known as demi-regularities [33, 57]. Byng and colleagues underline that the realist approach in evaluation does not explicitly discuss the importance of the interactions between mechanisms or feedback loops, whereas in the original realist writings of Bhaskar, they are seen as fundamental to emergence [45]. Furthermore, as Connelly recalls [3, 58], the importance of time is recognized in critical realism. Some users also stress the interplay between agency and structure. Social structures provide resources that enable agents to act, and agents are therefore able to transform social structures by responding creatively to the circumstances in which they find themselves [34].

### Clarity and precision of the concept mechanism

According to the Daigneault and Jacobs’ conceptual framework, a concept is clear when its term has just one sense within a given referent and it is that much more precise when the empirical phenomena to which it applies are clearly distinct from those to which it does not apply [20].

Firstly, some of the terms employed by users are an integral part of the lexicon of the realist approach as “power” and “force.” Wilson and McCormack confer upon the mechanism the power to generate outcomes [56], while Jagosh and colleagues see in it a generative force which leads to these outcomes [38]. However, the use of some terms reveals difficulties in a clear and precise comprehension of this concept with regard to the attributes presented earlier. For instance, few users assimilate mechanisms to intervention strategies or activities implemented [31, 58–60]. However, as recalled by Jagosh and colleagues [36, 38], mechanisms (i.e., reasoning and reactions) are linked to, but not synonymous with, the intervention strategies which are, according to these authors, intentional measures or a rational plan taken by program implementers.

Secondly, the concept of mechanism will become more precise and clearer once the frontier between the concepts of context and intervention has been clearly settled.

### Mechanism versus context

According to users, these two concepts can be confusing, the boundaries between them being blurred [55, 61]. Pawson and Tilley [9] associate the context with the “spatial and institutional locations of social situations together, crucially, with the norms, values, and interrelationships found in them” (p. 216). Wong and colleagues also support a similar view referring the context as the prevailing beliefs, social and cultural norms, regulations, and economic factors [53]. Pawson and Tilley [12] honed their definition describing context as being the characteristics of the conditions in which interventions are introduced (and this even prior to its implementation [47]). Corresponding to the “backdrop” of interventions [38], the concept of context is also useful to describe the pre-existing characteristics of the individuals, localities, situations, or systems of interpersonal and social relationships in which an intervention is being set up. As Robert and colleagues have reiterated, context is social, cultural, historical or institutional [33]. In a nutshell, quoting Pawson and colleagues [25, 26], Macfarlane and colleagues use the four layers of contextual factors that shape the implementation of the social programs: (1) the individual capabilities of the key actors to take the intervention forward (e.g., values, roles, knowledge, purpose); (2) the interpersonal relationships supporting the intervention (e.g., communication, collaboration, network, influences); (3) the institutional settings (e.g., informal rules, organizational culture, leadership, resource allocation, local priorities); and (4) the infra-structural system (e.g., political support) [62]. These contextual layers can thus be at micro-level (e.g., individual actors), meso-level (e.g., departments and teams), or macro-level (e.g., organization) [35]. So, describing context and its effects through constraining or enabling factors [56] is the in-depth examination of all these elements which could prove relevant for our understanding of mechanisms [45, 60, 63, 64]. So, context, by interacting with mechanisms through its constraining and enabling factors, determines the direction of outcomes and change [39, 52, 65]. That is why the relationship between these mechanisms and the effects of the context in which they exist need to be understood [56]. Acknowledging context and mechanisms can be used to modify program theory (i.e., logic of the intervention), can help to explain why the intervention worked or not in a certain context, can help to identify where the intervention is likely to be most effective [58], and can strengthen the implementation of an intervention to other contexts (similar or not).

### Mechanism versus intervention

Pawson and Tilley [12] remind us that the concept of mechanism sometimes becomes conflated with the one of intervention. In the realist approach, the attributes of the concept of mechanism are attached to the attributes of an intervention that is by nature complex and dynamic. As Pawson and Tilley said, the term mechanism is not used to distinguish the components of an intervention, each one of which will work through its own underlying processes (i.e., sequences of mechanisms) [12]. Whereas mechanisms (i.e., reasoning and reactions of human agents) refer to “the ways in which any one of the components of the intervention or any set of them, or any step of series of steps (e.g. decision-making steps) brings about change” [8] (p. 7), interventions can be seen as the “opportunities that an agent, situated inside structures and organizations, can choose to take” in a given context to bring about changes ([8], p. 62). As the scoping review reveals, the concepts of mechanism and intervention are located at different levels of abstraction. The attributes of the concept of intervention arise out of a more all-encompassing approach such as strategies and implemented activities whereas the attributes of the concept of mechanism seek to be more centered on the elements of individual or collective reasoning or reactions of agents in regard of the available resources allotted to the intervention implementation [25, 27]. Interventions can be regrouped around the mechanisms out of which they are built [24, 45, 53]. The realist approach focuses more on families of mechanisms rather than on families of interventions [53].

### The operationalization of the concept “mechanism”

With the scoping review, we found that users have tried to categorize mechanisms in order to unpack, define, and prioritize them. This resulting typology takes different forms, dependent on the way in which the logic of the intervention is understood. For instance, Marchal and colleagues differentiate vision (i.e., what the team wants), from discourse (i.e., what the team says) and the actual practice of the intervention (i.e., what the team does) [18]. As a result, their typology of mechanisms is structured according to the different levels of interpretation and analysis suggested: i.e., targeted causal mechanisms (which are, according to these authors, close to the level of vision), theorized causal mechanisms (discourse) and observed mechanisms (arising out of practice). Similarly, Ridde and Guichard have highlighted different types of mechanisms: theoretical mechanisms (i.e., mechanisms proposed prior to the study), candidate mechanisms (i.e., mechanisms empirically collected during the study), and confirmed mechanisms (i.e., mechanisms empirically confirmed by the study) [31].

## Discussion

Our scoping study of the concept of mechanism, assessed against the three dimensions of a concept identified by Daigneault and Jacobs [20], has yielded a rich picture of the evolving conceptual perspectives of both the proponents of the realist approach and of the users trying to enrich or operationalize the approach.

In the scoping study, only those writings featuring a definition of the concept of mechanism grounded in the realist approach were selected. The object of this study being this specific concept, we did not attempt at assessing the quality of the studies. In the same way, the analyses of the attributes of the concepts of context and intervention, presented above, are not exhaustive, given that these were not targeted by our inclusion criteria. These could, therefore, be the subject of a closer, more detailed study with a view to offering an exhaustive list of the attributes of these two concepts, both intimately related to that of the mechanism in the realist approach. However, given that initially, all the writings cited as references by Pawson and his colleagues have been read and analyzed through these different concepts; we can confidently say that the evidence collected remains solid and representative of what these concepts stand for in the realist approach.

### Understanding the relationships between mechanism, intervention, and context: focus on the realist ontology

Our work pinpoints various difficulties that evaluators will need to address in order to make it useable in their practice. First of all, the identification and categorization of mechanisms proves to be a real challenge. Some mechanisms, as they pre-date the intervention, will elude the designers of the intervention. Moreover, public health interventions being complex, they encompass several concomitant mechanisms which can operate in parallel. On top of that, one needs to take into account the temporality of mechanisms and their relations with context, intervention, and other mechanisms to produce outcomes. These elements appear essential in order to categorize them before and during implementation of the intervention and its evaluation.

Next, the realist ontology seems relevant for understanding the complex and dynamic nature of public health intervention within a realist evaluation. Pawson reminds us that “realism is a general research strategy rather than a strict technical procedure” [77] (p. 14). For Bhaskar [78], a critical realist quoted by several users [34, 45, 49, 56, 57], the reality is stratified into three levels: the real (including the causal mechanisms and intransitive structures, which pre-exist beyond the consciousness of—individual or collective—human agents), the actual (including the events produced when the causal mechanisms are activated), and the empirical (what reflexive agents experiment to understand the phenomena). “Structures and mechanisms are real and distinct from the patterns of events that they generate; just as events are real and distinct from the experiences in which they are apprehended” [78] (p. 56). A public health intervention stands therefore as an event in a system [79]. This intervention can be described by its fixed functions and its forms that can vary in different contexts [80]. In other words, the functions can be seen as essential mechanisms (e.g., the mechanisms for reducing the social inequalities of health could be one of them), and the forms as strategies and implemented activities resulting from the interaction between functions and context. Pre-existing context (or pre-intervention context) and context of action could be distinguished [79]. The three levels of reality (i.e., the real, the actual, and the empirical) are also useful to interpret and explore the interrelationships between structures, human agents, and mechanisms. Archer, another influential theorist in the critical realism movement, illustrates the interplay between structure and agency over time within the morphogenetic approach [81]. Even though human agents depend on structures and their resources, they can also transform these structures through their actions. The causal mechanisms emanate both from the individuals and the social relations and structures which they form. It is important to bear in mind that both structures and agents are elements of context. As put by Poland and colleagues [82], context can be seen as “the local mix of conditions and events, social agents, objects and interactions which characterize social systems, and whose unique confluence in time and space selectively activates, triggers, blocks or modifies causal powers and mechanisms in a chain of reactions that may result in very different outcomes depending on the dynamic interplay of conditions and mechanisms in time and space” (p. 309). So, mechanisms would be for Pawson and Tilley the way-of-reasoning and reactions of human (individual or collective) agent(s) to bring about changes through the implementation of an intervention according to the resources available in a given context.

One strategy to improve the understanding of the diversity of these mechanisms could pass by the identification of the elements pertaining to the domain of the following: the real (i.e., causal mechanisms as functions of the intervention), the actual (i.e., intervention strategies as forms of the intervention), and the empirical (i.e., data collected during the intervention evaluation). Applied throughout the different steps of the evaluation process (theory building and theory testing), this strategy would make more explicit the multiple interrelationships between the mechanism of interest and the contextual factors (e.g., characteristics of individuals or collective agents—stakeholders, practitioners, or participants—and structures—pre-existing resources and/or new resources implemented by the intervention) and the various elements of the intervention, such as time (programming or implementation processes), space (micro- or macro-level), and form (strategies and implemented activities).

### Definition of mechanism

Based on our thorough analysis of the literature on mechanism in a realist approach perspective, we can now suggest our own definition of this central concept that builds on Astbury and Leeuw’s [15].

#### A mechanism is hidden but real

To gain a better grip, and clarify the ontological focus chosen, the mechanism of interest should be characterized according to how it fits in the intervention and within the different strata of the social reality (i.e., the real, the actual, and the empirical). Existing prior to the intervention, but latent, a causal mechanism reveals itself during implementation of the intervention within a given context. Sensitive to the variations of context be they at the micro-, meso-, and macro-levels, it produces expected or unexpected outcomes that may or may not be favorable to a change in the problematic situation.

#### A mechanism is an element of reasoning and reactions of (an) individual or collective agent(s) in regard of the resources available in a given context to bring about changes through the implementation of an intervention

A mechanism results in the interaction between human agents, intervention, and structures. It reflects the logic of intervention of the various actors involved directly (e.g., stakeholders) or indirectly (e.g., populations) in the intervention.

#### A mechanism evolves within an open space-time and social system of relationships

A mechanism is dynamic and it may be interacting with other mechanisms (family of mechanisms), which may or may not be parallel, with the same process or another, with elements of context or with the effects it has itself produced (i.e., positive or negative feedback). The multiplicity and the temporality of mechanisms are thus important elements to be taken into account during the implementation and evaluation of an intervention. Indeed, interventions are subjected to the influence of other interventions (and thus to external mechanisms and contextual factors), have history of their own (sometimes that goes back well before their implementation), may also arise out of earlier interventions, and grow and evolve beyond their planned duration.

As one can readily see, this definition constitutes a refinement of the different dimensions of the key concept of mechanism and clarifies its attributes in order to facilitate its operationalization in public health program evaluation.

## Conclusion

Like any methodological innovation, the realist approach in evaluation is gradually built through the challenges arising during its operationalization. It therefore came as no surprise to see the definition of the concept of mechanism evolving [19], just like the concepts of context and intervention that are so intimately related to it. This study has been useful on two fronts: (1) the analysis we carried out has yielded a clearer, more precise definition of the concept of mechanism, drawing on what the attributes of the concept are, going beyond Astbury and Leeuw’s three attributes that are said to be realist [15] and (2) this review has also underlined the importance of specifying the various levels of intervention, through the dynamic of mechanisms in its multiform context, to bring about changes. As a complement of the recent articles published to better operationalize the realist approach [19, 83, 84], the results of this study may in turn trigger further improvements in the way the realist approach is applied in evaluative practice in public health and potentially beyond.

## Additional files

Additional file 1: **References of Pawson et al. included (1997-2012).** (PDF 214 kb) Additional file 2: **Documents (n=49) included in the scoping study (from 1997 to June, 2012).** (PDF 307 kb)
