# Supplementary material for: The concept of mechanism from a realist approach: a scoping review to facilitate its operationalization in public health program evaluation
Source: Implement Sci. 2015 Oct 30;10:153. doi: 10.1186/s13012-015-0345-7 (PMC4628377; doi:10.1186/s13012-015-0345-7)
Supplement: Additional file 1: — References of Pawson et al. included (1997-2012). (PDF 214 kb) [file 13012_2015_345_MOESM1_ESM.pdf]

Additional file 1: References of Pawson et al included (1997-2012)

Authors, Date	Type of document	Term of mechanism	Sense of mechanism	Referent of mechanism
Pawson R, Tilley N (1997) [9]	Book	Mechanism, Program mechanisms, Generative mechanism, Causal mechanisms, Potential program mechanisms, Causal powers, Explanatory mechanisms, Mechanism of change, Problem mechanism	'program mechanism': (1) Reflect the embeddedness of the program within the stratified nature of social reality, (2) Take the form of propositions which will provide an account of how both macro and micro processes constitute the program, (3) Demonstrate how program outputs follow from the stakeholders' choices (reasoning) and their capacity (resources) to put these into practice<br>The generative mechanisms thus actually constitute the regularity; they are the regularity.<br>A mechanism is thus not a variable but an account of the make-up, behaviour and interrelationships of those processes which are responsible for the regularity.<br>A mechanism is thus a theory; a theory which spells out the potential of human resources and reasoning.<br>Mechanisms refer to the choices and capacities which lead to regular patterns of social behaviour. Causal mechanisms are at work in generating those patterns of behaviour which are deemed 'social problems' and which are the rationale for a program.<br>Causal powers reside not in particular objects or individuals but in the social relations and organizational structures which they form.	**<u>Realism</u>**<br>Evaluation deals with real and the reality we seek to explore is stratified. Evaluation should follow a realist methodology (…) which suggests a more extensive role for theory (…) Evaluation needs to be realistic.<br>(…) realist tradition in the philosophy of science. Realists refer to the embeddedness of all human action within a wider range of social processes as the stratified nature of social reality.<br>**<u>Social interventions</u>**<br>Social interventions only and always work through the action of mechanisms, through a process of weaving resources and reasoning together.<br>Programs work by introducing new ideas and/or resources into an existing set of social relationships. Social programs are (…) social systems. They comprise (…) the interplays of individual and institution, of agency and structure, and of micro and macro social processes.<br>**<u>Theory-driven evaluation</u>** (Chen and Rossi, 1981; Chen, 1990): What we need to know is what it is about a program which makes it work.
Pawson R (2002) [24]	Peer-reviewed article	Mechanism, Programme mechanism, Intended mechanism, Causal powers of a	The causal power of an initiative lies in its underlying mechanism (M), namely its basic theory about how programme resources will influence the subject's actions. Whether this mechanism is actually triggered depends on context (C), the characteristics of both the subjects and the programme locality.	**<u>Realism</u>**<br>Realist inquiry has a long pedigree in social science<br>The realist approach is not an evaluation technique as such, but a framework for the whole enterprise. It is being developed to encompass programmes of all types (local to transnational) and evaluation tasks of

		programme, Underlying mechanism, Anticipated generative mechanism	It is not programmes that work but the resources they offer to enable their subjects to make them work. This process of how subjects interpret the intervention stratagem is known as the programme mechanism and it is the pivot around which realist evaluation revolves.<br>The intended mechanism is simply to make available a resource that the subjects do not possess and it will work if those subjects are persuaded to accept, install, maintain and act upon it.<br>Broadly speaking, the anticipated generative mechanism is that the incentive offers deprived subjects the wherewithal to partake in some activity beyond their normal means or outside their normal sphere of interest, which then prompts continued activity and thus long term benefit to themselves or their community.	all modes (developmental to impact assessment).<br>In the philosophy of science, the germ of the same idea lies at the base of Popper's (1959) theory of 'falsification'. (…) In other words falsification is a method of long-term knowledge resolution (Campbell, 1974).<br>**<u>Theory-driven evaluation</u>**<br>There is an obvious affinity here with the 'theory-driven' approaches to evaluation.
Pawson R, Tilley N (2004) [12]	Paper	Mechanism, Programme mechanism, Triggers of change, Programme incentives	Mechanisms describe what it is about programmes and interventions that bring about any effects. Mechanisms are often hidden.<br>Mechanisms also explain a programme's failure.<br>Mechanisms thus explicate the logic of an intervention: they trace the destiny of a programme theory; they pinpoint the way in which the resources on offer may permeate into the reasoning of the subjects.<br>In fact, it is not programmes that work but the resources they offer to enable their subjects to make them work. This process of how subjects interpret the intervention stratagem is known as the programme mechanism and it is the pivot around which realist evaluation revolves.<br>(…) 'mechanism' refers to the ways in which anyone of the components or any set of them, or any step of series of steps brings about change.	Realist evaluation is a species of **<u>theory-driven evaluation</u>**.<br>Realist evaluation is about theory testing and refinement.<br>(…) **<u>social programmes</u>** are regarded as products of the human imagination: they are hypothesis about social betterment. Programmes are thus shaped by a vision of change and they succeed or fail according to the veracity of that vision. Evaluation, by these lights, has the task of testing out the underlying programme theories.<br>According to realist evaluation programmes are 'theories incarnate', they are 'embedded in social systems', they are 'active', and they are parts of 'open systems'.<br>Programmes are complex social systems introduced amidst complex social systems. Both of these

			The triggers of change in most interventions are ultimately located in the reasoning and resources of those touched by the programme.<br>(…) mechanisms activated by the interventions will vary and will do so according to saliently different conditions.<br>Mechanisms are the engine of change in realist thinking and these describe how programme resources seek to influence their subject's<br>reasoning.	systems are open. Programmes are composed of an intricate, reverberating sequence of decisions, which will shift this way and that over the duration of the programme. The systems in which they are introduced – organisations, localities, welfare regimes, moral communities, belief configurations – are also in permanent transition. Furthermore, the relationship between the two systems is dynamic. A powerful programme can alter the conditions that made it work in the first place, thus changing its effectiveness over time<br>**Realism**, however, goes a step further in understanding the changing nature of programmes. That is to say, they are regarded as self-transformational. Successful interventions can change the conditions that made them work in the first place.<br>(…) realists regard programmes as rather sophisticated social interactions set amidst a complex social reality.<br>Realist inquiry can be located in every social science discipline. Given this miscellany of topics, it should be clear that realism is not a research technique as such. It is a 'logic of inquiry' that generates distinctive research strategies and designs.<br>Realism's vision of programmes as configurations of theories provides a rationale that may be helpful in formulating that strategy.<br>Realism rejects the particular formulation arguing that programmes are active, and thus it is the operation of particular mechanisms acting in context that brings about change.<br>Realism operates at middle range, using concepts

				that describe interventions at a level between the big policy ideas and the day-to-day realities of implementation.
Pawson R, Greenhalgh T, Harvey G, Walshe K (2005) [26]	Peer-reviewed article	Mechanism<br>Underlying mechanisms<br>Working mechanism<br>Potential mechanism<br>Underpinning mechanism of action	The success of an intervention thus depends on the cumulative success of the entire sequence of these mechanisms as the programme unfolds.	**<u>Realism:</u>** What is it about this programme that works for whom in what circumstances?<br>**<u>Complex interventions:</u>** (1) complex interventions are theories, (2) such interventions are active, (3) intervention theories have a long journey, (4) their implementation chains are non-linear and can even go into reverse, (5) interventions are fragile creatures, embedded in multiple social systems, (6) they are leaky and prone to be borrowed, (7) they are open systems that feedback on themselves.
Pawson R (2006) [25]	Book	Mechanism<br>Generative mechanisms<br>Causal mechanisms<br>Generic mechanism	Mechanisms are the engines of explanation in realist analysis. We can make rough sense of the world through its demi-regularities.<br>Mechanisms explain causal relations by describing the 'power' inherent in a system, be those (…) agents (like policy-makers) or structures (like social programmes). The structure generates the work pattern.<br>The mechanism explains what it is about the system that makes things happen.<br>For the realist, causal explanation cannot begin without the identification of generative mechanisms (…) The prerequisite is to look beneath the surface in order to inspect how they work. The development of cumulative knowledge about 'what works' requires sustained investigation of the generic mechanism, namely the operation of choices under the inducement of programme resources.	**<u>Realism</u>** is solidly placed to supply a durable understanding of the process of cumulation of social scientific knowledge.<br>Realism is a methodological orientation, or a broad logic of inquiry that is grounded in the philosophy of science and social science.<br>Critical realism is not one jot of use to us here because its leap into the arms of the normative (…) Accordingly, the realism pursued here is the other sort, the one without the adjective.<br>Campbell (…) emphasized the importance of theory, both in manufacturing the data and in explaining the observed regularities. Campbell thus sits between positivism and relativism. He was a realist in that he accepted the existence of a reality which is independent of our senses, but which we can only discover through our senses.<br>It is the 'critical' element that causes the confusion. In a nutshell, it is realist in exploiting the principles laid down in early realist philosophy of science, and

				the working tools established in the application of that philosophy to evaluation methodology. It is critical realist only in its understanding of the quarrelsome process through which scientists approach objectivity (…)<br>(…) realism stands foursquare behind the generative model of causation.<br>**Social interventions:** (1) Interventions are theories, (2) Interventions are active, (3) Intervention chains are long and thickly populated, (4) Intervention chains are non-linear and sometimes go into reverse, (5) Interventions are embedded in multiple social systems, (6) Interventions are leaky and prone to be borrowed, (7) Interventions are open systems and change the conditions that make them work in the first place.
Pawson R, Wong G, Owen L (2011) [27]	Peer-reviewed article	Mechanism	/	**Realist evaluation** (Henry, Julnes, and Mark, 1998)<br>**Theory-driven evaluation** (Weiss, 1995)<br>**Complex Social Programs:** (1) Programs are active, not passive, (2) Programs have long implementation chains and multiple stakeholders, (3) Programs are embedded in complex social systems, (4) Programs are implemented amid the turbulence of other interventions, (5) Programs beg, steal, borrow, and adapt, (6) Programs are the offspring of previous interventions, (7) Programs change the conditions that make them work in the first place.
Pawson R, Manzano-Santaella A (2012) [28]	Peer-reviewed article	Mechanism<br>Programme mechanisms<br>Underlying mechanisms<br>Particular	In all cases, the outcome patterns come to be as they are because of the collective, constrained choices of all stakeholders.<br>In all cases, investigation needs to understand these underlying mechanisms in order to capitalize on the gains accrued in charting the differential effectiveness	**Realism** is a general research strategy rather than a strict technical procedure. It has always been stressed that innovation in realist research design will be required to tackle a widening array of policies and programmes. It has always been stressed that this version of realism is Popperian and Campbellian

		mechanism<br>Incentive	of the intervention.<br>Mechanisms are embodied in the subjects' reasoning and they are best investigated therein.<br>Programme mechanisms change minds. They open eyes.<br>The action of a particular mechanism in a particular context will generate a particular outcome pattern.<br>In realist terminology, there will always be multiple Ms – a proliferation of ideas within a programme, creating different resources that trigger different reactions among participants.<br>Under realism, mechanisms are not regarded as the multiple components of combined interventions. Rather mechanisms penetrate to the layer beneath, attempting to explain how particular measures work.<br>Programme mechanisms capture the many different ways in which the resources on offer may impinge on the stakeholders' reasoning.	in its philosophy of science and thus relishes the use of the brave conjecture and the application of judgement. From these vantage points, it can be seen that realist inquiry is a broad and welcoming church.<br>Realist evaluation has its own slogan – 'what works for whom in what circumstances… and why'.<br>The core realist intention has always been to complement the measurement of change by understanding how it is generated (Archer, 1998). By throwing light into the black box we understand the transformation of input to output.<br>(..) a realist investigation will hypothesize, monitor and seek to explain how the same programme resource is interpreted and acted upon in different ways by different participants in different positions.<br>Realist evaluation is avowedly **theory-driven**; it searches for and refines explanations of programme effectiveness.<br>(…) the phrase 'theory-driven' (…) designs that attempt to utilize the realist explanatory apparatus without a prior grounding in programme theory will end with explanations that are ad hoc and piecemeal.
