# Supplementary material for: The concept of mechanism from a realist approach: a scoping review to facilitate its operationalization in public health program evaluation
Source: Implement Sci. 2015 Oct 30;10:153. doi: 10.1186/s13012-015-0345-7 (PMC4628377; doi:10.1186/s13012-015-0345-7)
Supplement: Additional file 2: — Documents (n=49) included in the scoping study (from 1997 to June, 2012). (PDF 307 kb) [file 13012_2015_345_MOESM2_ESM.pdf]

Additional file 2: Documents (n=49) included in the scoping study (from 1997 to June, 2012)

Authors, Date	Type of document	Realist approach	Area of practice	Term of mechanism	Sense of mechanism	Referent of mechanism
Blaise & Kegels, 2004 [29]	Peer-reviewed article	Realist evaluation	Health system management	mechanism	In a nutshell, a mechanism produces an outcome in a given context. Applied in a different context, the same mechanism will produce expected and unexpected, even adverse effects (p.340).	
Wong et al, 2012 [53]	Peer-reviewed article	Realist framework	Medical teaching	mechanism, programme mechanism, generative mechanism, potential mechanism	A mechanism may be usefully defined as: "…underlying entities, processes, or [social] structures which operate in particular contexts to generate outcomes of interest". Certain contexts in the social world around us 'trigger' mechanisms to generate outcomes (sometimes abbreviated to CMO).<br>Mechanisms (…) are not 'visible', but must be inferred from the observable data; they are context-sensitive, and they generate outcomes (p.91).<br>Programme mechanisms can be identified by asking what it is about a programme that generates change (p.92).<br>The mechanisms (…) are often multiple, that some mechanisms are obvious and correspond to those intended by the course designers, and that some are less obvious and are unanticipated by the designers. (...) A mechanism is not inherent to the intervention, but is a function of the participants and the context (p.92).<br>They cannot be seen or measured directly (because they happen in people's heads); they are context-sensitive; they are multiple (hence, when researched, they need to be unpicked, defined and prioritised) (p.92-93).	**Realism** middle-range theories (i.e. theories that are constructed as part of a realist synthesis or realist evaluation) will be built around one or more mechanisms, but will involve more than just the mechanisms (i.e. they will also involve C and O) (p.93).<br>In realism, it is mechanisms that trigger change rather than interventions themselves and thus realist reviews focus on "families of mechanisms" rather than on "families of interventions" (p.93-94).
Kane et al, 2010 [46]	Peer-reviewed article	Realist synthesis	Health care services	Mechanism, negative mechanism	The term 'mechanisms' (…) refers to the reactions, triggered by the intervention within a certain context, which lead to a certain outcome.	Mechanism is the pivot around which **realist enquiry** revolves.

Marchal et al, 2010 [18]	Peer-reviewed article	Realist evaluation	Health system management	Mechanism, underlying mechanism	If human action is embedded within a wider range of social processes and structures, then causal mechanisms reside in social relations and context as much as in individuals (p.2).<br>The mechanism is the causal pathway that explains how the intervention leads to an observed outcome in a particular context. In other words, the intervention leads to an outcome in specific contexts if it triggers certain mechanisms (p.12).<br>The mechanism (…) be context-dependent (p.12).	We described the **<u>intervention</u>** in terms of content and application, and the intended and actual outcomes. We drew on our interviews and observations to differentiate (proclaimed) vision (what the team wants), the discourse (what they say) and the actual practices (what they do).
Robert et al, 2012 [33]	Peer-reviewed article	Realist synthesis	Health care systems	mechanism	An intervention does not work in itself; it is the mechanisms that underlie it which act (or fail to act) to produce the observed outcomes. These mechanisms are influenced by the context in which the intervention is implemented (p.3).<br>Mechanisms should be understood as elements from the reasoning of actors facing interventions (p.4).<br>A mechanism: (1) is generally hidden, (2) is sensitive to context variations and (3) produces outcomes (p.4).	We must understand how the agent and the structure interact to produce what characteristics **<u>social reality</u>**.<br>As social systems, **<u>social interventions</u>** are built from the interplay between agents and structure.
Holma & Kontinen, 2011 [41]	Peer-reviewed article	Realist evaluation	NGO	mechanism, generative mechanism, change mechanism, structural mechanisms, causal power, causal mechanisms, programme mechanisms	The concept of generative mechanism focuses exactly on the question of HOW, in a certain context, the outcomes are generated (p.186).<br>In terms of evaluation approach, (…) analytical attention regarding the generative mechanism is focused both on the individual's reasoning, and on the collective capabilities and resources that enable or hinder different generative mechanisms (PT 1997) (p.186).<br>(…) a range of change mechanisms (…) depending on the programme context, the subjects and the locality. The concept of mechanism is relevant to understanding how problematic situations are generated and what type of alternative mechanisms	The position of the **<u>realist approach</u>** between constructivism and positivism implies that certain mechanisms beyond individuals' perceptions and interpretations are assumed, but simultaneously, with regard to the social world, it is emphasized that such mechanisms are not immune to actors' interpretation and transformation.

					the interventions provide. Moreover, this concept guides evaluators to investigate how beneficiaries interpret interventions, and how they use the new resources (p.190).	
Ridde & Guichard, 2011 [31]	Peer-reviewed article	Realist evaluation	Health promotion/ Prevention	mécanisme, mécanisme candidat, mécanisme candidat confirmé, mécanisme potentiel ou théorique	La notion de mécanisme a été ici comprise plus au sens des stratégies mises en œuvre que des attitudes et réactions des acteurs, telle que l'approche de pawson et Tilley le suggère.<br>Les mécanismes mis au jour doivent encore être compris comme des mécanismes "candidats". Cependant, le fait que certains confirment ce que la seule recherche à notre connaissance avait relevé comme étant des mécanismes candidats, nous permet de les qualifier de mécanismes candidats confirmés. En revanche, d'autres mécanismes apparus dans la présente recherche n'était pas connus dans la recherche, nouveaux mécanismes candidats qui viennent confirmer empiriquement les mécanismes théoriques. Enfin, certains mécanismes préalablement notés n'ont pas été retrouvés dans les actions françaises analysées. Pour ces derniers, conserve le statut de mécanismes candidats.	
Lhussier et al, 2011 [59]	Peer-reviewed article	Realist framework	Health promotion/ Prevention	potential mechanism, intervention mechanism	Activities/interventions<br>(…) the intervention they (practitioners) deliver<br>(…) interventions anticipated to be the most effective and so acceptable in the context. / Intervention implementation strategy / intervention set up.	
Hoddinott et al, 2010 [52]	Peer-reviewed article	Realist framework	Health promotion/ Prevention	underlying causal mechanism	Underlying causal mechanisms, although they may not be directly observable, are nonetheless real (p.771)<br>This would parallel the components of realist evaluation, where the mechanisms that have causative associations with the outcomes are situated within a contextual circle (p.772).	

Pommier et al, 2010 [32]	Peer-reviewed article	Realist evaluation	Health promotion/ Prevention	Mechanism Action mechanisms	A mechanism is "not a variable but an account of the make-up, behavior and interrelationships" of the processes which are responsible for the change, "a mechanism is thus a theory" (p.2).	They [Pawson & Tilley] considered a **program** to be a system of assumptions (i.e. action mechanisms leading to expected outcomes) that the evaluation process tests to develop a theory that can be applied and amended, for example, for the same program in different contexts.
Jackson & Kolla, 2012 [60]	Peer-reviewed article	Realist evaluation	Health care services	mechanism	The intervention components or mechanisms (p.2) The mechanisms were activities or actions taken in the program (p.5)	
Jagosh et al, 2011 [36]	Peer-reviewed article	Realist synthesis	Public health research	mechanism	Mechanism pertains to what 'turns on' the minds of program participants and stakeholders that make them want to (for example) participate or invest in programs. They may be cognitive or emotional responses, typically in relation to program resources being offered. Mechanisms are not synonymous to program strategies, which are intentional measures taken by program implementers. Identifying mechanisms advances the synthesis beyond describing 'what happened' to theorizing 'why it happened, for whom, and under what circumstances' based on participant reasoning or reaction (p.7).	
Macaulay et al, 2011 [65]	Peer-reviewed article	Realist synthesis	Public health research	program mechanism	Program mechanism usually refers to the mediating variables in the change process, but here refers more specifically to the resources offered by the program and the reasoning of the participants who choose or choose not to participate (p.46-47).	Although these concepts have long histories in the health and social science literature, here they (context, mechanism, and outcome) are defined in terms of their usefulness for **programme theory** testing.
Rycroft-Malone et al, 2011 [35]	Peer-reviewed article	Realist evaluation	Public health research	implementation mechanism, sub-mechanism	(…) implementation mechanisms and sub-mechanisms, with associated enabling and constraining factors, which included networks (hard and soft), evidence, structures, contracts,	**Complex social interventions** according to Pawson and Tilley are comprised of theories, involve the actions of people, consist of a chain of

					gouvernance and roles (p.4).	steps or processes that interact and are rarely linear, are embedded in social systems, prone to modification and exist in open, dynamic systems that change through learning. Programmes are broken down so that we can identify what it is about them (mechanisms) that might produce a change (impact), and which contextual conditions (context) are necessary to sustain changes.
Wong et al, 2011 [73]	Peer-reviewed article	Realist synthesis	Health promotion/ Prevention	mechanism	The mechanism behind their motivation for such levels of supports (p.6).	We would then aim to provide an explanation of this **demi-regularity** through the identification mechanism(s) (p.4). **Complex social interventions** - where outcomes arise in a non-linear way thought multiple human interactions and decisions under the influence of a myriad of contextual factors.
Robert, 2011 [40]	Report	Realist framework	Health care services	mécanisme cognitif	Les mécanismes cognitifs (ou attitudes des acteurs) / Un mécanisme cognitif est un comportement ou un raisonnement, individuel ou collectif, qui se révèle lors de la mise en place d'une intervention (p.222).	
Byng et al, 2005 [45]	Peer-reviewed article	Realist evaluation	Health care services	potential mechanism of causation, positive and countervailing mechanisms, active mechanisms	The potential mechanisms of causation residing in both actors and society are real and present even when not active, and when actualized may or may not be observable (empirical). Whether or not an outcome or regularity occurs is determined by the interplay of positive and countervailing mechanisms (p.72). (...) the mechanisms within the intervention were dependent on the local contexts in which they	**Critical realists** recognize the importance of both individual agency and the influence of the structures and culture of society (p.72). Realist evaluation does not explicitly discuss the importance of the interactions between mechanisms or feedback loops, whereas in the original realist writings of Bhaskar

					operated (p.73).<br>Mechanisms could be positive, absent or negative.<br>(…) each mechanism or context involving social structures, human action and thought is itself made up of many CMO configurations when the psychological, then physiological and biochemical processes are considered (…) Can a visible activity such as a case discussion be considered a mechanism, when surely the aim of RE is to 'surface' mechanisms? Our response is that it depends on the level of social reality that one is engaged in (p.89).<br>(…) the mechanism being the series of actions required to (…)	(1998) and others they are seen as fundamental to emergence.<br>Whereas Pawson & Tilley tend to focus interest on one mechanism and search for contingent contexts, Bhaskar and Archer talk more of additional mechanisms, which may be active or countervailing (p.90).<br>The **<u>intervention</u>** was based on a belief that the systems involved are complex and holistic -in the sense of involving layers of reality - and that these layers of interest are real if not always tangible.
Byng et al, 2008 [39]	Peer-reviewed article	Realist evaluation	Health care services	mechanism, external mechanism, key mechanisms	(…) assigning preplanned and emergent components of the MHL intervention and other significant external occurrences (such as a PCT audit) as mechanisms (p.4).<br>Mechanisms, both part of the intervention (eg elements of facilitation) and external influences (p.4).<br>Mechanisms: flexible and fixed components of the intervention.	In order to develop '**<u>middle range theories</u>**' about how they lead to outcomes
Pittam et al, 2010 [43]	Peer-reviewed article	Realist evaluation	Health care services	mechanism, emerging mechanism, interacting mechanisms	(…) mechanism is the way in which an intervention works within the given context to produce a particular outcome or change (p.599).<br>Mechanisms - exploring the processes set in motion by the intervention (p.600).	Realist evaluation is based on the idea that behind the workings of a particular programme or intervention there is an underlying theory which explains change (p.599)
Pedersen et al, 2012 [55]	Peer-reviewed article	Realist evaluation	Health promotion/ Prevention	mechanism, underlying mechanism	The underlying mechanism(s) M connect(s) (the intervention) X and outcome O, and the context C in which the relationship(s) occurs (…) Context and mechanisms are seen as the factors which initiate or trigger the causal relationships; for instance motivation from the key actors is a necessary	Acknowledging context and mechanisms, such as role behaviour by key actors as a necessary ingredient for the program to work, can be used to modify program theory. It can also help explain why

					ingredient for the program to work (p.49).<br>Mechanisms: role behaviour, motivation and trust in the key actors affects outcome. Mechanisms are defined as relevant personal characteristics of key actors or interpersonal relations between them.	the program worked or not in a certain context, and to identify where the intervention is likely to be most effective (p.51).<br>Occupational safety **interventions** are complex social activities, set in complex, dynamic social systems. The probability of an intervention succeeding is therefore crucially dependent on how the organizational context and personal characteristics of key actors affects the implementation of the intervention.
Rycroft-Malone et al, 2012 [57]	Peer-reviewed article	Realist synthesis	Health research	Mechanism, mechanisms of action, underlying causal mechanism, mechanisms underlying implementation activity, particular actions of change	(…) the mechanisms by which an intervention works (or fails to work)<br>The premise is that in certain contexts individuals are likely (although not always certain) to make similar choices, and therefore particular contexts influence our choices such that reoccurring patterns emerge, i.e., demi-regularities.<br>It was agreed that an intervention was a broad concept, while strategies referred to mechanisms or approaches to achieve the intervention.	(…) to uncover the underlying theories that explain these **demi-regularities** by examining the interactions between mechanism, context, and outcome. These questions were framed as 'what' questions in order to help us determine what it is about the particular actions (mechanisms) of change agents that have an impact and connect themes to action.<br>Implementation of such interventions is often accompanied by complex strategies comprising support structures, resources and processes.<br>For a realist synthesis, an **intervention** is a theory; because interventions are implemented on a hypothesis of if we do X in this way, then it will bring about an outcome.<br>It was agreed that an intervention was

						a broad concept, while strategies referred to mechanisms or approaches to achieve the intervention.
Wand et al, 2011 [49]	Peer-reviewed article	Realist evaluation	Health care services	Mechanism, key mechanisms	Mechanisms refer to people's choices, decisions and capacities, describing how people react when faced with an intervention and it is the pivot around which realistic evaluation revolves (p.202).	A realistic evaluation framework was applied to identify key "mechanisms" triggered by the intervention with the aim of generating a set of "**middle range theories**" (p.199)<br>Identifying mechanisms involves the attempt to develop propositions about what it is within the program that triggers a reaction from its subjects. Health care interventions only work through the action of mechanisms, through a process of weaving resources and reasoning together (p.202).<br>A **critical realist** standpoint contends that all programs involve interplay between individual and institution, between social structures and human agency, of micro and macro social processes and that all programs also involve disagreement and power play. Realistic evaluation asserts that an intervention or program is likely to activate multiple mechanisms with divergent outcomes.
Wand et al, 2010 [34]	Peer-reviewed article	Critical realism + realist evaluation	Health care services	generative mechanism, underlying mechanism,	Generative mechanisms refer to the structures, powers and relations that explain how a programme or intervention works beneath the surface. Although not directly observable, generative mechanisms are	**Critical realists** recognise the stratified or multilayered nature of the social world and that causal mechanisms operate at different strata or levels or

				mechanism	real and can be identified through their effects.<br>Generative mechanisms may remain latent until they are activated under specific circumstances (p.233-234).<br>According to realistic evaluation, the causal power of a programme lies in the underlying mechanisms and in this basis theory of how programme resources will influence outcomes in a particular context (p.234).<br>This pathway from resource to reasoning is referred to as the programme 'mechanisms' (p.235).<br>Mechanisms are understood from the context in which they were born and which illuminates them (p.235).<br>Mechanisms capture people's reasoning and their choices and describe how people react when faced with a policy measure or intervention (p.235).	reality (p.234).<br>The interplay between structure and agency is emphasised in critical realism. Social structures provide resources that enable individuals to act, as well as placing limits on individual behaviour. The behaviour of human agents, however, is not exclusively determined by social structures. Agents are therefore able to transform social structures by responding creatively to the circumstances in which they find themselves (p.234).<br>A critique of the prevailing social order acknowledges the ability of human actors to identify and challenge health inequalities and sources of oppression (p.234).<br>The CMO configuration operationalises the main tenets of critical realism, acknowledging the relationship between the social and natural world, the multilayered nature of the social world, the interaction between social structures, mechanisms and human agency, and that any programme must be understood within the context in which it is delivered.<br>For realist evaluation-based research, **programmes** are not monoliths, people are not passive recipients of

						opportunities to improve their health and context is pivotal to understanding the interplay between programmes and their effects. People are the critical factor in any intervention in a social science context and it is people that cause the programme to work, not the programme itself. This is based on the premise that if people are provided with certain resources, it may change their thinking and behavior.
Ranmuthugala et al, 2011 [44]	Peer-reviewed article	Realist evaluation	Health care services	mechanism, enabling mechanism, disabling mechanism, mechanism of change, key mechanism	Mechanisms will be defined as the factors and resources that the CoP offerts its members to influence a change in behaviour or action. A mechanism may be an enabler or a disabler depending on the context (p.3).	
Porter & O'Halloran, 2012 [66]	Peer-reviewed article	Critical realism + realist evaluation	Health research	Mechanism, causal mechanism, generative mechanism	It assumes that the mechanisms introduced by the intervention are not the only ones in operation, so to understand the outcomes observed, it is necessary to identify the mechanisms pertaining and the nature of their interaction. Its aim is to discover if, how and why interventions have the potential to cause beneficial change (p.19).<br>(…) a complex chain of reasoning and reaction, or more usually, a number of chains (causal mechanisms in realist terms) (…) In different contexts, different mechanisms or combinations of mechanisms will pertain (p.22).<br>(…) the causal mechanisms inhibiting or promoting	These are most notable when we consider **complex** healthcare **interventions** which rely on chains of human reasoning and action.

					the implementation and sustainability of the LCP (p.22)<br>(…) combinations of mechanisms affecting the implementation of the LCP (p.22)<br>(…) the generative powers of a specific mechanism, it cannot tell us of the outcomes resulting from those powers in an open system, which will be dependent upon the relationship of that generative mechanism with the other mechanisms at paly within any given context (p.26)	
Rycroft-Malone et al, 2010 [61]	Peer-reviewed article	Realist evaluation	Clinical practice	causal mechanism, mechanism, mechanism of action	Because causal mechanisms are always embedded within particular contexts and social processes, there is a need to understand the complex relationship between these mechanisms and the effect has on their operationalisation and outcome. (p.2)<br>Mechanisms are theories, which are based on a hypothesis or proposition that postulates... if we deliver a programme in this way or we manage services like this, then we will bring about some improved outcome. Mechanisms are contingent upon contexts (p.3).	Protocol-based care, a **complex intervention** in itself, was being studied within the complex system of health care delivery consisting of layers of actors, social processes, and structures.
Ogrinc & Batalden, 2009 [63]	Peer-reviewed article	Realist evaluation	Medical teaching	mechanism	Mechanisms are the processes through which the intervention is implemented. For a social or educational clinical improvement project, this includes individual interpretations. Mechanisms focus on how an intervention works, and often the mechanisms are hidden from (or obscured from) those who observe the outcome patterns (p.663).<br>Mechanisms are the true facts or 'cause-effect' statements of how the system operates (p663)<br>Mechanism describes what it is about the interventions that bring about effects. The process(es) through which subjects interpret and act on the	Mechanism is the pivot around which **realist research** revolves (p.663).<br>In social change, **interventions** are considered "theory incarnate".<br>Complex systems require regular monitoring and evaluation to improve them, for complexity is marked by adaptable interactions that behave in non-linear ways.

					intervention.	
Wong et al, 2010 [75]	Peer-reviewed article	Realist synthesis	Medical teaching	mechanism	Mechanims are processes operating within an intervention that describes how the 'human components' use the resources available to them (p.2).	**<u>Middle range theory</u>**
Oroviogoicoeche & Watson, 2009 [64]	Peer-reviewed article	Realist evaluation	Clinical practice	mechanism	In Realistic Evaluation, "the main aspect is that of generative mechanisms based on causality being, not external, but an internal potential of the programme or intervention that is activated in the right conditions" (p.841).<br>The reasons and resources to enable users to obtain the outcomes and it is the actions of the different stakeholders that makes the intervention system works (p.841)	
Rycroft-Malone et al, 2008 [67]	Peer-reviewed article	Realist evaluation	Clinical practice	Mechanism, causal mechanism	Mechanisms: appropriate ideas and opportunities to groups / practices they prompt (p.868)	
Kastner et al, 2011 [37]	Peer-reviewed article	Realist synthesis	Clinical practice	Mechanism, underlying mechanisms, causal mechanism, mechanisms of implementability	Causal mechanisms: theorie of change<br>The underlying mechanism (M) that connects them (O and two events X and Y) and the context (C) in which the relationship occurs (p3)	
McCormack et al, 2007 [74]	Peer-reviewed article	Realist synthesis	Clinical practice	Mechanism, actual mechanism, causal mechanism	Plural forms of evidence that are generated through the complex interactions between processes (mechanisms) (p.8)<br>Causal mechanisms, operating in particular circumstances, create certain change (p.8)	**<u>Complex social interventions</u>** comprise theories, involve the actions of people, consist of a chain of steps or processes that interact and are rarely linear, are embedded in social systems, are prone to modification and exist in open systems that change through learning.

Leone, 2008 [70]	Peer-reviewed article	Realist evaluation	Health promotion/ Prevention	Mechanism, underlying mechanisms	Mechanisms ‘developed in the field resulting from the interactions between the actions of the initiative and the reactions of the target groups’. According to realistic evaluation, the causal power of the programme lies in its underlying mechanism and in its basic theory about how programme ressources will influence outcomes (O) (e.g. a subject’s behavior) in a particular context (C) (p.10).<br>These are activated as a consequence of the interaction between the programme and particular subgroups of users (p.18).<br>The sequence of mechanisms (…) had been subject to changes: in fact, during implementation, the pilot programme brought out new unforeseen and sometimes unwelcome outcomes (p.16).<br>Different reactions of the individuals towards a programme (undesired and desired reactions). (p.18).<br>In terms of mechanisms we have identified how the operators have interpreted their task (…) and the reactions of the recipients (p.24).<br>The sequence of mechanisms activated in the programme partly corresponds to the sequence hypothesized by the decision-makers at the outset; while parallel mechanisms have emerged, which affect the total outcomes in both negative and positive values.<br>Concept of 'family of mechanisms' (Pawson 2006) (p.27)	Thanks to the concept of 'mechanism', we attempted to explain 'how' the subjects reacted to similar interventions developed in several contexts, and why different effects were observed (p.26).
Dieleman et al, 2009 [47]	Peer-reviewed article	Realist synthesis	Health system management	mechanism, mechanism for change	Mechanisms : how they worked / or processes / mechanisms for change / mechanisms=outputs<br>Mechanisms that triggered change were increased knowledge and skills, feeling obliged to change and health workers' motivation.	

Marchal et al, 2010 [30]	Peer-reviewed article	Realist evaluation	Health system management	mechanism, underlying mechanism of change, generative mechanism	The mechanisms that underlie such practices, or in other words the ways in which such bundles work (p.2).<br>A realist evaluation examines the generative mechanisms that underlie an intervention at micro- and macro-level, and explores how actors' choices and use of their resources led to the outcome. It also focuses on the embeddedness of the intervention in the social reality (p.4)<br>"the underlying mechanism between intervention and outcome (p.3)	**Middle-range theorie**<br>We described the **intervention** in terms of content and application, and the intended and actual outcomes. We drew on our interviews and observations to differentiate (proclaimed) vision (what the team wants), the discourse (what they say) and the actual practices (what they do).
Maluka et al, 2011 [48]	Peer-reviewed article	Realist framework	Health system management	mechanism, mechanism of change	Mechanisms: the stakeholders' ideas about how the change will be achieved through an intervention (p.4)	**Interventions** that seek to influence change in this type of context are generally complex and dynamic; often evolving in response to local circumstances, target-group engagement and other events beyond the control of the implementers, which can adversely affect the impact of the intervention.
Macfarlane et al, 2011 [62]	Peer-reviewed article	Realist evaluation	Health system management	mechanism	A realist approach to case study focuses particularly on identifying the mechanisms (or theories of change) that are in policymakers' minds as they pursue particular change efforts, and on asking how and why the same underlying change mechanism has different contexts (p.59).	
Tolson et al, 2007 [54]	Peer-reviewed article	Realist evaluation	Health system management	Mechanism, mechanism observed / conjectured	In this study, a mechanism might be educational outreach	**Realism** recognizes that the world is an open system, with layers of structures that interact to form mechanisms and contexts (p186)<br>The interactions between context (workplace challenges) and mechanisms (facilitation processes)

						that served to create positive or negative feedback. Negative feedback reduced the likelihood of success, positive feedback facilitated change (p.193).
Evans & Killoran, 2000 [68]	Peer-reviewed article	Realist evaluation	Health promotion/ Prevention	Mechanism, project mechanism	Mechanisms: processes or resources (p.128)	In this conceptualization, **social programmes** work by introducing new ideas, processes or resources (i.e. mechanisms) into existing social relations (i.e. context).
Clark et al, 2005 [42]	Peer-reviewed article	Realist framework	Health promotion/ Prevention	Mechanism, social mechanism	Mechanisms: choices and capacities they (programs) offer in different circumstances (p364) / individuals' reactions to it under different circumstances and at different times (p.364).	
Greenhalgh et al, 2009 [50]	Peer-reviewed article	Realist evaluation	Health system management	mechanism, mechanism of change, submechanisms , underlying mechanisms	Mechanisms: the stakeholders' ideas about how change will be achieved in an intervention (p396) (…) mechanisms of change were hard to detect at the front line and in real time and that when some participants offered candidate mechanisms, others would often contest them (p.412).	
Keller et al, 2010 [71]	Peer-reviewed article	Realist evaluation	Health care innovation	mechanism	what mechanisms (e.g. individual, organizational, cultural) will enable the reactions or changes (p.13). Mechanisms explain causal relations by describing the "powers" inherent in the system (p.14). The mechanism explains what it is about the system that makes things happen.	
Jagosh et al, 2012 [38]	Peer-reviewed article	Realist synthesis	Public health research	mechanism	A mechanism is the generative force that leads to outcomes. It often but not always denotes the reasoning (cognitive or emotional) of the various actors in relation to the work, challenges, and successes of the partnership. Mechanisms are linked to, but not synonymous with,	**Middle-range theorie**

					the program's strategies (e.g., a strategy may be a rational plan, but a mechanism involves the participants' display of responses to the availability of incentives or other resources) (p.8).	
Wilson & McCormack, 2006 [56]	Peer-reviewed article	Realist evaluation	Health research	Causal mechanisms, mechanism for change	(…) by describing the mechanisms and structures through which various often unobservable entities possess the "power" to generate outcomes (p.47).<br>Because causal mechanisms always occur in a particular social context, the relationship between these mechanisms and the effects of the context in which they exist needs to be understood. Causal mechanisms operate within constraining or enabling factors (the effects of the contexts) (p.47).<br>"the causal mechanisms within the environment, any interactions that occur between mechanisms and the power of mechanisms to generate outcomes" (p.47).<br>The same mechanism can produce different outcomes dependent on the context (p.48).<br>This means a programme (or initiative) includes all the players, the venue, its past, and its history.<br>Mechanisms for change are formed by modifying the capacities, resources, constraints, and choices facing both participants and practitioners. The relationship between the mechanism and the outcome is dependent upon the context of location and social norms, i.e where it takes place and how the rules of that place influence the mechanism and outcome (p.51).	**<u>Critical realism</u>**<br>Causal mechanisms (domain 1 - real) can neutralize one another in such a way that no event takes place (domain 2 - actual), and there is no observable change in reality (domain 3 - empirical) (p.47).<br>Pawson and Tilley (1997) developed the following formula to represent this: Context+Mechanism=Outcome.<br>This means a **<u>programme</u>** (or initiative) includes all the players, the venue, its past, and its history. // The programme relies on the actors' insights and preferences.
Koenig, 2009 [72]	Peer-reviewed article	Realist evaluation	Evaluation	Underlying mechanisms, generative mechanisms, mechanism	The causal power of an initiative lies in its underlying mechanism (M). Whether this mechanism is actually triggered depends on context (C). A given policy or measure is liable to activate multiple mechanisms with divergent outcomes (O) and similar outcomes	**<u>Programmes</u>** are theories.<br>(…) the vital ingredients of programme ontology are its 'generative mechanisms' and its 'contiguous context'.

					may, in principle, be generated through varying mechanisms (p.25).	
Michielsen et al, 2011 [76]	Peer-reviewed article	Realist synthesis	Health care services	mechanism	Whether these underlying mechanisms are actually triggered, and produce the desired outcomes, depends largely on the given context and the characteristics of the subjects (p.2).	
Ogilvie et al, 2011 [69]	Peer-reviewed article	Realist evaluation	Health promotion/ Prevention	Mechanism, causal mechanism, postulated mechanism	(…) how particular patterns of outcomes may be produced by particular causal mechanisms being triggered in particular contexts (p.479)	Realist evaluation involves developing theories about how particular patterns of outcomes may be produced by particular causal mechanisms being triggered in particular contexts (so-called context, mechanism, and outcome configurations) and testing these theories cumulatively across different instances of the interventions.
Connelly, 2007 [58]	Peer-reviewed article	Realist framework	Health promotion/ Prevention	Generative mechanism, mechanism at work, specific mechanism, mechanism	The initiation of causal sequences can be traced to the working of (triggering of) 'generative mechanisms'. The triggering of a specific mechanism depends on the contextual factors being of the correct kind (...) Given a generative mechanism (M) and a conductive context (C) for its triggering, we can expect to see and measure specific observations (O) and events (p.936). (…) the mechanism or mechanisms to be invoked (triggered) or blocked, and the distribution of such mechanisms, so who will be affected, where, when and so on (p.940).	The importance of time is recognized in **critical realism** by what Archer calls the morphogenetic approach. This approach recommends a historical orientation to the understanding of a given context and an extant distribution of positions and power (p936).<br>(…) the mechanisms at work in the given context are investigated. This stage will draw on large-range theories or concepts as well as local work using epidemiological and qualitative methods, both oriented to building a theory of causation by the detailed description of generative mechanisms. This will allow further

						work with the population group to imagine or identify (historically) efficacious mechanisms for countering the injustice or ill health-generating circumstances.<br>This means an attempt to uncover generative mechanisms operating at the level of the real; the enquiry aims to delve beyond the simply empirical and through an actualism (observable regularity) to the level of the real (p.938)
Steven et al, 2007 [51]	Peer-reviewed article	Realist evaluation	Interprofessional education	Mechanism	Mechanisms included the content of the sessions and the procedures involved (p.251)<br>The term “mechanisms” encompasses the processes and interactions that take place when an initiative is put into practice. The concept of “mechanism” works both on the level of “surface (observable) appearance” and “inner (hidden) workings”. The relationship between mechanisms (…) and their effects (…) is not fixed but contingent on the context (p.253)	
